# Cryptic Determinant of α4β7 Binding in the V2 Loop of HIV-1 gp120

**DOI:** 10.1371/journal.pone.0108446

**Published:** 2014-09-29

**Authors:** Boonrat Tassaneetrithep, Doreen Tivon, James Swetnam, Nicos Karasavvas, Nelson L. Michael, Jerome H. Kim, Mary Marovich, Timothy Cardozo

**Affiliations:** 1 Office for Research and Development, Faculty of Medicine Siriraj Hospital, Mahidol University, Bangkok, Thailand; 2 United States Military Health Research Program, Walter Reed Army Institute of Research, Silver Spring, Maryland, United States of America; 3 Department of Biochemistry and Molecular Pharmacology, New York University School of Medicine, New York, New York, United States of America; 4 Department of Retrovirology, United States Army Medical Component, Armed Forces Research Institute of Medical Sciences, Bangkok Thailand; Emory University School of Medicine, United States of America

## Abstract

The peptide segment of the second variable loop of HIV-1 spanning positions 166–181 harbors two functionally important sites. The first, spanning positions 179–181, engages the human α4β7 integrin receptor which is involved in T-cell gut-homing and may play a role in human immunodeficiency virus (HIV)-host cell interactions. The second, at positions 166–178, is a major target of anti-V2 antibodies elicited by the ALVAC/AIDSVAX vaccine used in the RV144 clinical trial. Notably, these two sites are directly adjacent, but do not overlap. Here, we report the identity of a second determinant of α4β7 binding located at positions 170–172 of the V2 loop. This segment – tripeptide QRV^170–172^– is located within the second site, yet functionally affects the first site. The absence of this segment abrogates α4β7 binding in peptides bearing the same sequence from position 173–185 as the V2 loops of the RV144 vaccines. However, peptides exhibiting V2 loop sequences from heterologous HIV-1 strains that include this QRV^170–172^ motif bind the α4β7 receptor on cells. Therefore, the peptide segment at positions 166–178 of the V2 loop of HIV-1 viruses appears to harbor a cryptic determinant of α4β7 binding. Prior studies show that the anti-V2 antibody response elicited by the RV144 vaccine, along with immune pressure inferred from a sieve analysis, is directed to this same region of the V2 loop. Accordingly, the anti-V2 antibodies that apparently reduced the risk of infection in the RV144 trial may have functioned by blocking α4β7-mediated HIV-host cell interactions via this cryptic determinant.

## Introduction

**Figure 1:**
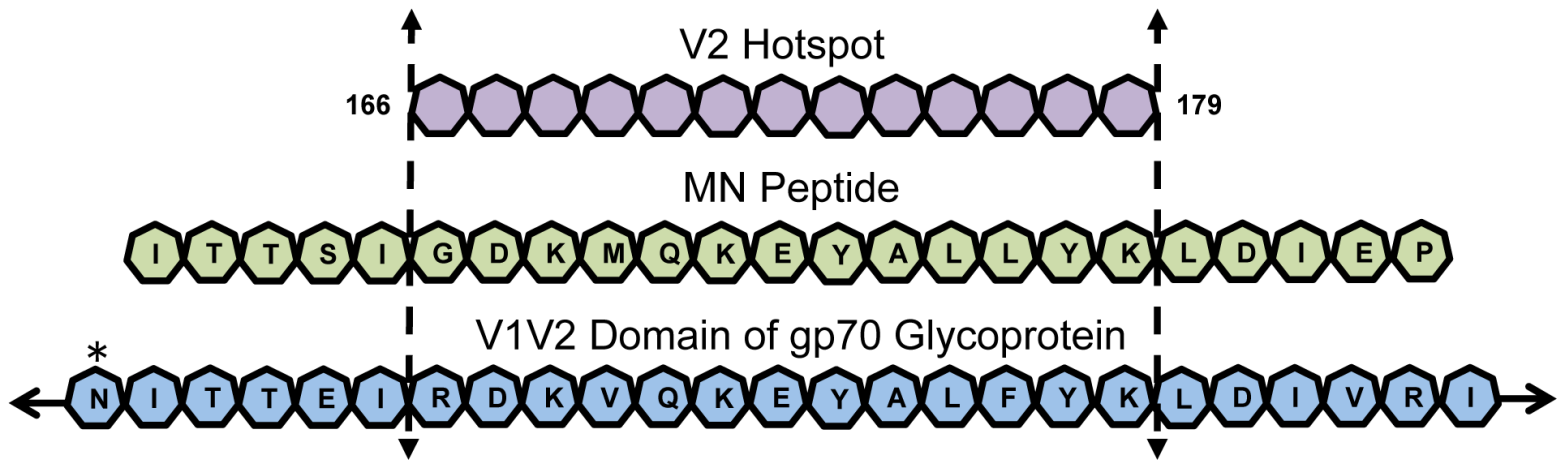
Protection maps to positions 166–179 of the V2 loop. Of the approximate 270 assays performed in the RV144 immune correlates analysis, only Abs binding three reagents showed an OR of 0.5 or lower. These reagents were the gp70-V1V2 glycoprotein (sequence for a portion of the V1V2 domain of gp70 shown in blue heptagons with glycosylation sites indicated by black stars), the MN peptide (sequence shown as green heptagons from position 161–183), and the V2 Hotspot (shown as purple heptagons spanning positions 166–179). All three of these reagents include an unglycosylated portion of the V2 domain spanning positions 166–179.

In the RV144 study, HIV infection was reduced by 31.2% [1]. A subsequent immune correlates analysis revealed that high titers of vaccine-elicited antibodies (Abs) directed against the V1/V2 domain of the surface envelope glycoprotein (gp120) of the HIV virus were associated with a significantly lower odds ratio (OR) for infective events [2]. Secondary analysis revealed that Abs directed at a V2 peptide from the MN strain of HIV and directed at overlapping peptides in a microarray from the 166–178 region of the V2 loop were also associated with low ORs [3]. Out of approximately 270 assays in the immune correlates analysis, including viral neutralization assays, only serum Ab binding to three V1V2 domain derived peptides showed an OR of 0.5 or lower [3]. Interestingly, the common element among these three protection associated molecules is the peptide segment from positions 166–178 of the V2 loop (V2^166–178^; **Figure 1**). Independently, a recent sieve analysis of the RV144 trial compared viral sequences in infected volunteers and showed that subjects who were both vaccinated and infected with HIV lacked virus strains with a lysine at position 169 (K169) of the V2 loop [4], which was present in the vaccine immunogen. These data suggest that protective antibodies elicited by the vaccine and specific for K169 may have filtered out strains bearing a K169 in their V2 loops, indicating that immune pressure derived from the vaccine was directed at V2^166–178^. Thus, V2^166–178^ appears to harbor some epitopes targeted by protective Abs from the RV144 study.

The V2 loop has been shown to harbor a binding site for the human α4β7 integrin receptor [5], though the role this potential interaction plays in HIV-1 infection has been disputed [6]. This receptor was shown to be associated with dissemination of HIV to gut-associated lymphoid tissue (GALT), which was postulated to be important in the establishment and maintenance of HIV infection [7]. Subsequently, a non-human primate study showed that blocking α4β7 with a specific monoclonal antibody during a high-dose (200 50% tissue culture-infective doses) SIV infection decreased plasma viral load, gut tissue viral loads and proviral DNA in the GALT [8]. More recently, gp120-mediated signaling through α4β7 was reported to initiate the B-cell dysfunction commonly observed in HIV-infected subjects [9]. The binding site of the HIV virus to α4β7 is a tripeptide with an amino acid sequence typical for canonical integrin binding motifs. This tripeptide spans positions 179–181 of the V2 loop and most commonly consists of leucine-aspartate-isoleucine, or with isoleucine replaced by valine (LD[I/V]^179–181^). Recently, the anti-V2 Ab response elicited by the AIDSVAX-ALVAC vaccine used in RV144 was shown to map specifically to the segment at positions 166–178 of the V2 loop (V2^166–178^) [3], [10] with a strong dependence on amino acids at positions 169 and 172. Notably, this excludes the α4β7 binding site. Though these Abs were shown to be elicited by and targeted to V2^166–178^, they were not shown to neutralize HIV infection in *in vitro* CD4-mediated HIV infection neutralization assays and have not been tested for neutralization of α4β7-mediated homing to GALT [2]. These findings raise the question as to the function of the protective anti-V2 Abs in the RV144 trial. One possibility is that the Abs are non-neutralizing of either CD4-based or α4β7-based HIV-host engagement and instead, function via Fc-mediated functions such as antibody-dependent cellular cytotoxicity (ADCC) or complement fixation [11]. Alternatively, the Abs are neutralizing, but only neutralize α4β7-mediated functions and are therefore inactive in or invisible to the classical CD4-mediated neutralization assays that were performed in the RV144 immune correlates analysis. While the lack of overlap of the immunogenic V2^166–178^ with the α4β7 site speaks against the second possibility, these protective RV144 Abs may influence α4β7-mediated function by steric hindrance of α4β7 receptor access to the LD[I/V]^179–181^ tripeptide without binding LD[I/V]^179–181^ directly. The final possibility is that there is both a functional and a structural linkage between amino acids 170–172 within V2^166–178^ and LD[I/V]^179–181^ consistent with data reported here.

## Methods

### Primary α4β7^+^ T Cells Preparation

Frozen PBMCs – collected from healthy volunteers under an internal review board (IRB)-approved protocol, RV229/WRAIR number 1386– were thawed in complete media, counted, and checked for viability. CD4^+^ and CD8^+^ T cells were isolated from PBMCs by negative selection using Dynal magnetic beads following manufacturer guidelines (Invitrogen). Phenotyping was performed for purity and to confirm expression patterns (**Figure S1**). Primary CD4^+^ T cells and CD8^+^ T cells were cultured in the presence of 5 µg anti-CD3/anti-CD28, 10 nM all-trans retinoic acid and 20 IU rhIL-2. In some assays, the α4β7^+^ human B lymphoma cell line, RPMI8866 (Sigma), was used. All α4β7 expression levels were assessed using an anti-α4β7-APC conjugated monoclonal antibody (ACT-1, kindly gifted by Dr. A. Ansari), detected with an LSR II flow cytometer (Becton Dickinson) and analyzed with FlowJo 9.2 software.

### α4β7 Cellular Binding Assay

α4β7^+^ cells were plated at 200,000 cells per well on a 96-well plate and washed twice with binding buffer (10 mM HEPES, 150 mM NaCl, 1 mM MnCl_2_, 0.1 mM CaCl_2_, 0.5% BSA, 0.09% NaN_3_). V2 peptides or clade A gp120 (isolated from an infected patient in Uganda, submission to GenBank in process; kindly gifted by Dr. J. Arthos), clade A/E gp120 (CM244, RV254.006), clade C gp145 (CO6980v0.c22; kindly gifted by Dr. V. Polonis) were used at 2–5 µg final concentrations – after biotinylation according to manufacturer’s protocol (Thermo Scientific). Peptides or proteins were added to the cells, incubated for 30 minutes on ice, washed twice with binding buffer, then stained with β7 PE-Cy5 (BD Bioscience). The cells were then incubated with NeutrAvidin PE (Invitrogen) for 20 minutes at 4°C, washed twice with binding buffer, and fixed with 2% PFA/PBS. In some experiments, 4 mM EDTA was added prior to the peptide. The binding was determined using a LSR II flow cytometer (Becton Dickinson) and FlowJo 9.2 software as above.

### Diverse Peptide Selection

Four diverse peptide V2 sequences, representing the V2 segment from positions 165–185 and heterologous to the V2 sequences of the RV144 immunogens, were selected from HIV-1 strains of all subtypes from group M deposited at the Los Alamos National Laboratories (LANL) HIV Compendium. The sequences of the linear V2 peptides are shown in **Table 1**, where the integrin-binding motif in each of the linear peptides is in blue. Peptide 1 was selected as the 165–185 V2 segment from a strain with the most charged and polar amino acids among circulating strains, strain QB585.2102M.Ev1v5.C from clade A (**Figure S2**). Peptide 2 is the V2 loop crown sequence that occurs most commonly in recorded circulating strains, strain 878v3_2475 from clade B. Peptide 3 was selected as the V2 loop crown sequence, found in circulating strains with the most polar and charged amino acids and also exhibiting the most common V2 length of 40 amino acids, strain 01TZA341 from clade A. Peptide 4 represents the published consensus sequence for V2 165–185. Peptides were chemically synthesized and biotinylated at their N-termini by Genemed Synthesis, Inc. San Antonio, TX.

**Table 1:** Diverse and representative peptide set.

Peptide	Sequence	Length	Details	Strain/Clade	Genebank ID	Origin
1	LRDKK**QRV**YSLFYK**LDV**VQIN	21 amino acids	Highest polarity V2 most soluble	QB585.2102M.Ev1v5.C Clade A	ACS26794	Kenya
2	IRDKV**QKE**YALFYK**LDV**VPID	21 amino acids	Most commonly occurring	878v3_2475Clade B	EU184191	US
3	LRDKK**QQV**YSLFYR**LDI**EKIN	21 amino acids	Highest polarity of most common length	01TZA341Clade A	AY253314	Tanzania
4	IRDKK**QKE**YALFYK**LDV**VPID	21 amino acids	Published consensus V2	N/A	N/A	N/A

The sequences of the four peptides selected to represent the V2 loop region for a variety of HIV-1 viruses. Peptide 1 represents the most polar V2 peptide, Peptide 2 the most commonly occurring peptide, Peptide 3 the most polar peptide of the most common length, and Peptide 4 the published consensus V2 peptide.

### Sequence Alignments

**Figure 2:**
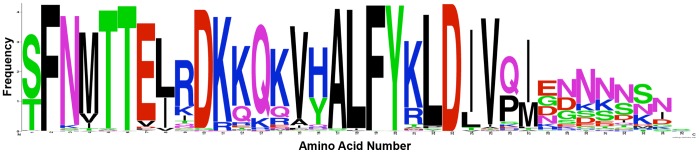
Alignment of V2 sequences. A logo indicating the conservation pattern of the amino acids comprising the V2 loop from position 158 to position 192. 464 V2 sequences from subtype AE viruses were aligned, with larger letters indicating higher conservation. Hydrophobic amino acids are indicated by black letters, charged amino acids by blue (+) and red (-) letters and polar amino acids are colored pink or green. Valine at position 172 is highly conserved in this population (numbering is according the Hxbc2 reference sequence by convention [21]).

All known V2 loop sequences from the Los Alamos National Laboratories Database (30830 sequences) were filtered to select only one sequence per patient (leaving 4200 sequences). This set of sequences was aligned using Clustal W, and subalignments corresponding to subtypes AE and B were provided to WebLogo (http://weblogo.berkeley.edu) to generate **Figure 2**.

### Peptide Folding

*Ab initio* folding of short peptides was done using the software ICM Pro (Molsoft LLC, La Jolla, CA) as previously described [12], [13]. Briefly, we built the peptide’s 3D atomic structure according to its sequence. We then set the simulation parameters to optimize accurate folding. These parameters include the number of free variables, the number of search steps per each local minimum, the length of the simulation, the temperature, the minimum gradient and the probability distribution. We then ran the simulation using a biased probability Monte Carlo procedure that generates random independent conformations of the peptide according to a predetermined continuous probability distribution. Each random conformation is then subject to a local minimization. Conformations are scored according to a number of factors including van der Waals energy, the internal energy of the peptide, hydrogen bonding energy, electrostatic energy, solvation energy, and entropic energy. All simulations were done using Molsoft ICM 3.7-2d. *Ab initio* folding results in an energy scored ensemble of conformations. The overall conformational preference of the folded peptide is best described by an energy-weighted average of the occurrence of specific conformations. In our case, we calculated the energy-weighted propensity of the peptide to form α-helical turns, which we call the “helicity” of the peptide’s conformational ensemble.

### Trimer Model

We used the published crystal structure of the BG505 strain trimeric gp140 spike (Protein Data Bank accession code 4nco). We modeled the two missing loops using the ICM Pro software as described previously [6]. One of these loops includes the α4β7 binding site (LD[I/V]^179–181^). We also changed K171 to arginine *in silico* in the structural model to illustrate where QRV^170–172^ would be located on the trimer.

## Results

**Figure 3:**
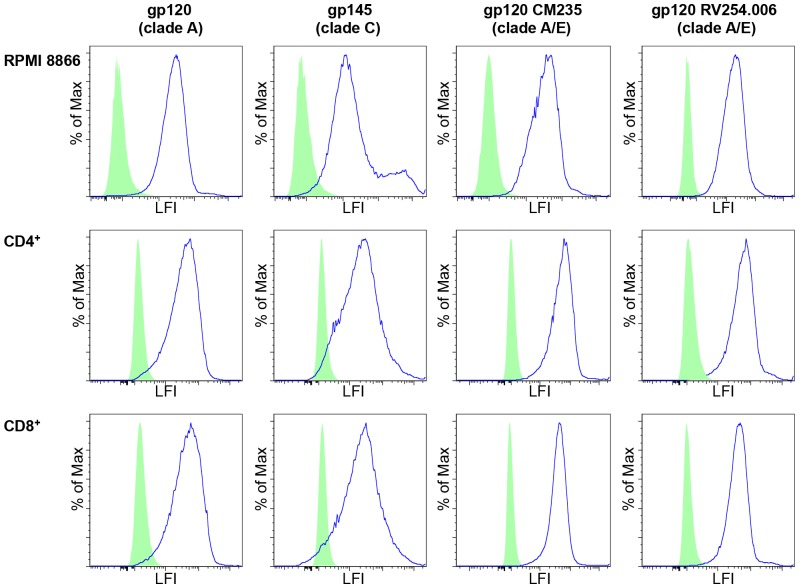
gp120 and gp145 bind α4β7 expressing cells. The blue histograms show binding of biotin labeled variants of gp120 and biotin labeled gp145 to activated primary CD4^+^ T cells, CD8^+^ T cells and the RPMI 8866 cell line expressing α4β7. Each row represents a different cell type: row 1) RPMI 8866 cells; row 2) CD4^+^ T cells; row 3) CD8^+^ T cells. Each column represents a different ligand: column 1) gp120 of clade A; column 2) gp145 of clade C; column 3) gp120 (CM235) Clade A/E; column 4) gp120 (RV254.006) clade A/E. The green histogram shows background binding to the NeutraAvidin negative control, LFI = Log Fluorescence Intensity.

**Figure 4:**
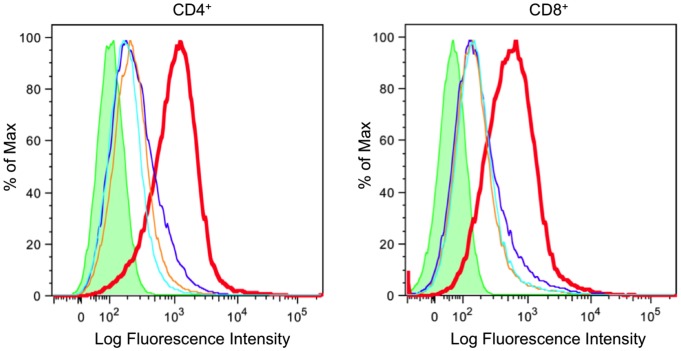
Specific monoclonal antibodies inhibit gp120 binding at protein or integrin level. CD4^+^ cells (left) and CD8^+^ cells (right) were incubated with biotinylated gp120 from clade A either without antibody pretreatment, red line (control) or after pretreatment with 2 µg of anti-integrin α4 mAb, HP2/1, (blue line), anti-V2 mAb 697D (orange line) or anti-V2 mAb 2158 (aqua). Binding was detected with NeutrAvadin PE and analyzed by FACS. The binding of clade A gp120 (red line) is inhibited by the addition of 2 µg anti-α4 mAb (blue line), anti-V2 mAb 697D (orange line) and anti-V2 mAb 2158 (aqua line). Green histogram is NeutrAvidin control, LFI = Log Fluorescence Intensity.

**Figure 5:**
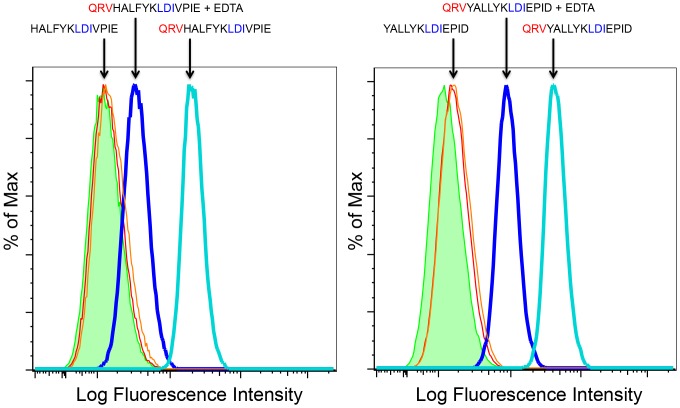
V2 derived peptides suggest a second determinant of integrin α4β7 binding. Biotinylated peptides representing the region around the canonical α4β7 tripeptide binding site (LD[I/V]^179–181^) in HIV strains A244 subtype AE (^173^HALFYKLDIVPIE^185^; red line, left panel) and MN subtype B (^173^YALLYKLDIEPID^185^;red line, right panel) were incubated with RPMI 8866 cells and detected with NeutrAvidin PE. The overlap with the control (green histogram) indicates no α4β7-binding. The aqua lines in both panels show the signal obtained when the QRV tripeptide is added to the N terminus of each of the original peptides. The blue line (both panels) indicates the effect of adding EDTA during incubation. Green histogram is NeutrAvidin control, LFI = Log Fluorescence Intensity.

We adapted an *in vitro* cellular assay [5] to measure the interaction of peptides with the human α4β7 integrin receptor expressed on the surface of primary CD4^+^ T cells, CD8^+^ T cells and the RPMI 8866 B cell line. This assay correctly and consistently detected the specific binding of gp120 variants and gp145 to the cell surface of α4β7 expressing cells (**Figure 3**). Further, the specificity of binding to α4β7 was demonstrated by the blocking antibody HP2/1 (Chemicon), that reduced gp120 binding to control levels (**Figure 4**, orange line). Binding of gp120 was also eliminated with the addition of anti-V2 antibodies (**Figure 4**, blue and aqua lines). This demonstrated that binding was dependent on positions 173–185 of the V2 region that was recognized by the antibodies. We further investigated how short V2 loop derived peptides containing the α4β7 site bound to the cell surface of primary CD4^+^ T cells and CD8^+^ T cells expressing the active form of α4β7. These N-terminal biotinylated short peptides, which span positions 173–185 of the V2 loop, mimic the exact sequence of the AIDSVAX immunogens from subtype AE (which is identical in this region for both strain TH023 used in the ALVAC canarypox prime and A244 which was part of the AIDSVAX protein immunogen) and subtype B (strain MN, a second component of the AIDSVAX protein immunogen). In both cases, the short peptides - despite containing the α4β7 binding site (LDI^179–181^) - did not bind the α4β7 integrin on the T cells tested as shown by the red lines in both the right and left panels of **Figure 5**.

A previously published analysis of V2 loop immunogenicity in the RV144 trial determined that changing a valine (V) to a glutamate (E) at position 172 abolishes the binding of RV144 vaccinee serum IgG to V2 loop peptides [10]. We aligned V2 sequences from subtype AE (described in the Methods section), the predominant circulating strain of HIV-1 in the RV144 trial, and found that Q^170^ and V^172^ are highly conserved in subtype AE (**Figure 2**). There is also some conservation for position 171 with a preference for a lysine (K), glutamine (Q), or an arginine (R). Adding an N-terminal tripeptide representing the consensus subtype AE sequence QRV at positions 170–172 of the V2 loop (QRV^170–172^) restored α4β7 binding to both the A244/TH023 (aqua line, **Figure 5**, left panel) and the MN strain V2^173–185^ peptides (aqua line, **Figure 5**, right panel). Finally, addition of EDTA to the assay greatly reduced peptide binding (blue line, **Figure 5**, left and right). Since integrin binding is dependent on Mn^2+^/Mg^2+^ ions, this confirms that the binding of these V2-derived peptides is α4β7 dependent.

**Figure 6:**
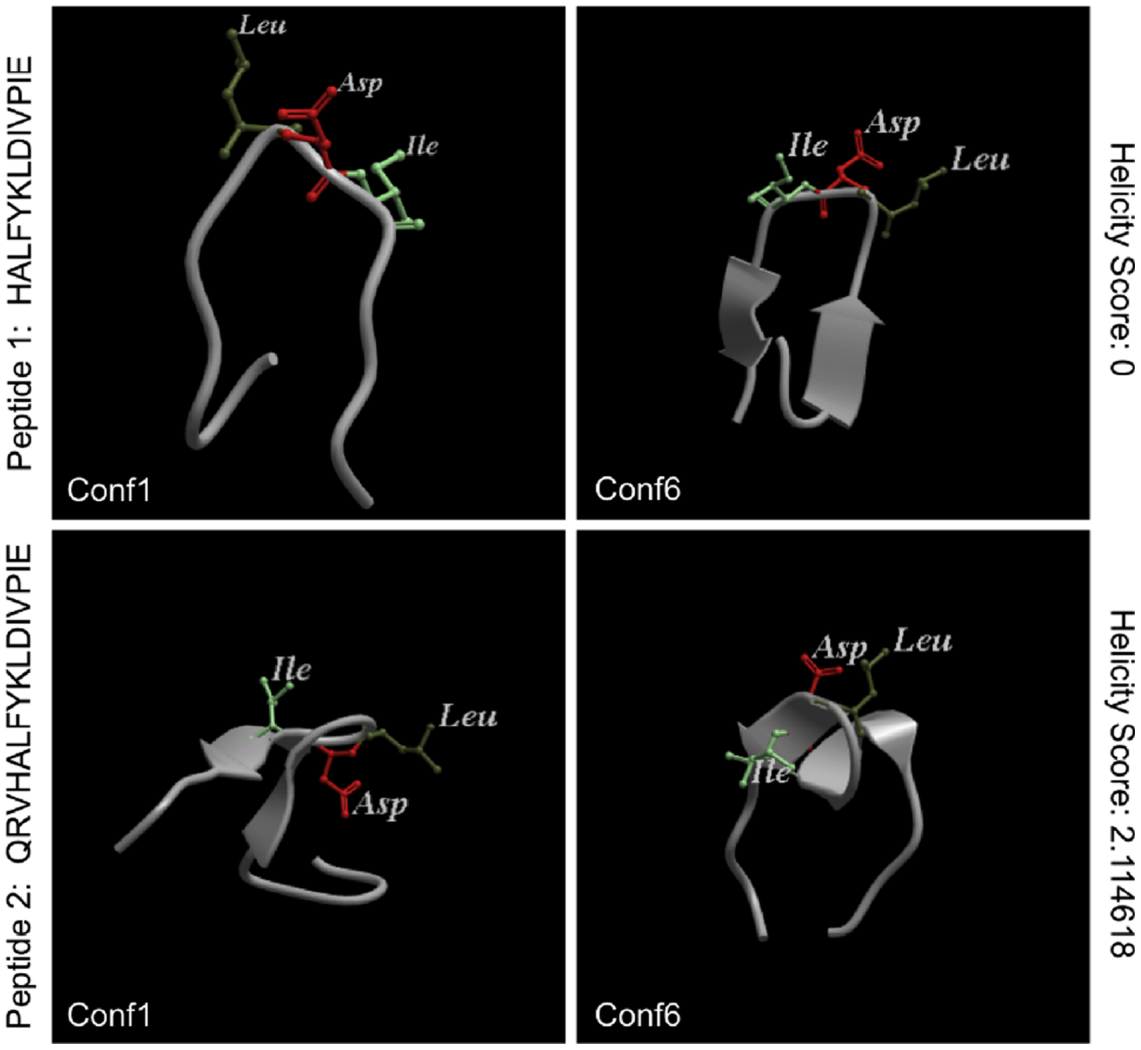
*ab initio* peptide folding. Peptide 1 (the sequence used in strain A244 of the RV144 vaccine) is predicted to consistently fold into a beta hairpin (upper panels). However, with the addition of QRV to the N-terminus of the peptide, the peptide is predicted to fold into more variable conformations including beta-like and alpha helical-like folds (lower panels). Conformations 1 and 6 of the 50 conformations generated by our software are shown. The peptide is shown in ribbon representation with the α4β7 binding domain (LDI^179–181^) shown in ball-and stick and colored according to residue.

In order to reveal structure-activity relationships, we folded the peptides used in this study *ab initio* in order to determine their conformational preferences. Small peptides are unlikely to have a fixed conformation, and their activity is more likely a function of a dynamic ensemble of their conformations. *Ab initio* folding can reveal these conformational ensembles for HIV variable loop peptides, perhaps in better detail than crystallography or NMR, as shown in several previous studies on HIV variable loop peptides [13], [14]. The addition of QRV changes the predicted conformation of V2 peptides generated by *ab initio* folding simulations. The A244 V2 peptide excluding QRV consistently folds into a beta sheet for all 50 of the most energetically favorable conformations (conformations 1 and 6 displayed in **Figure 6**; conformations 1–16 in **Figure S3**). However, the addition of QRV^170–172^ distorts the peptide into more variable folds such as partial beta sheets or alpha helices with highly favorable energies (**Figure 6** **, Figure S4**). We calculated the helicity score – a measure of the helical residues within a peptide – of the peptide with and without QRV and found the addition of the QRV tripeptide significantly changed the helicity score from 0 to 2.114618.

**Figure 7:**
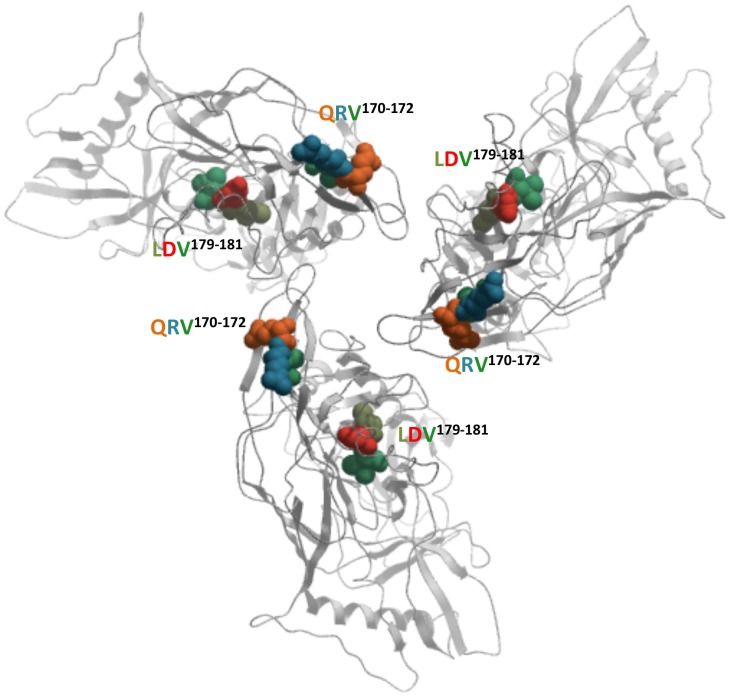
gp120 trimer showing linked functional sites. A representation of the QRV^170–172^ functional site in relation to the LDV^179–181^ α4β7 binding site. Residues shown in sphere representation and colored by residue type (glutamine = orange; arginine = cyan; valine = green; leucine = olive; aspartate = red). Protein shown in ribbon representation. This model was built using PDB 4nco as a template.

A recently published crystal structure of the gp140 trimer [15] provides a partial quaternary conformation for V2^166–178^. This structural model does not exhibit a conformation from V2 residues 178 to 190 due to lack of electron density. We modeled the conformation of the V2^166–178^ segment, including LDV^179–181^ and QRV^170–172^ within the published trimer structure (**Figure 7**). V2^166–178^ exhibits a β-strand conformation through position 177 that is highly stabilized by the tertiary structure of the overall V1V2 domain. Positions 178–190 emerge from the folded V1V2 domain and appear to be progressively unconstrained by surrounding atoms as one moves from N- to C-terminus of this segment.

**Figure 8:**
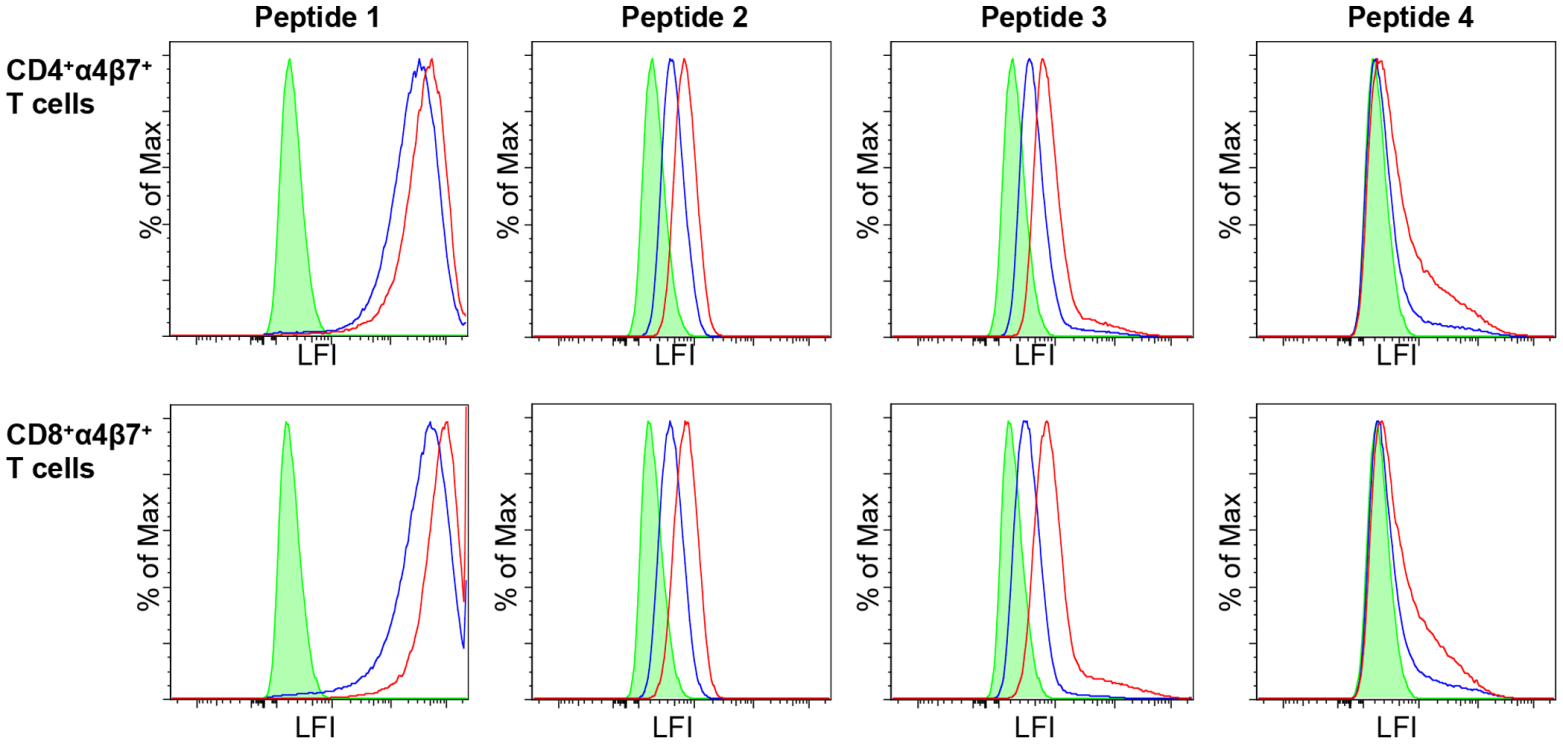
α4β7 diverse peptide binding assay. The binding of the representative set of 4 diverse peptides listed in Table 1 to α4β7 on CD4^+^ T cells (top row) and α4β7 on CD8^+^ T cells (bottom row). All peptides were tested at both 2 µg (blue line) and 5 µg (red line). In both cell lines, Peptide 1, the most polar and soluble peptide, showed maximal binding, while Peptides 2 and 3 showed submaximal binding. Peptide 4 showed no reactivity in either primary cell type. Green histogram is NeutrAvidin control, LFI = Log Fluorescence Intensity.

**Figure 9:**
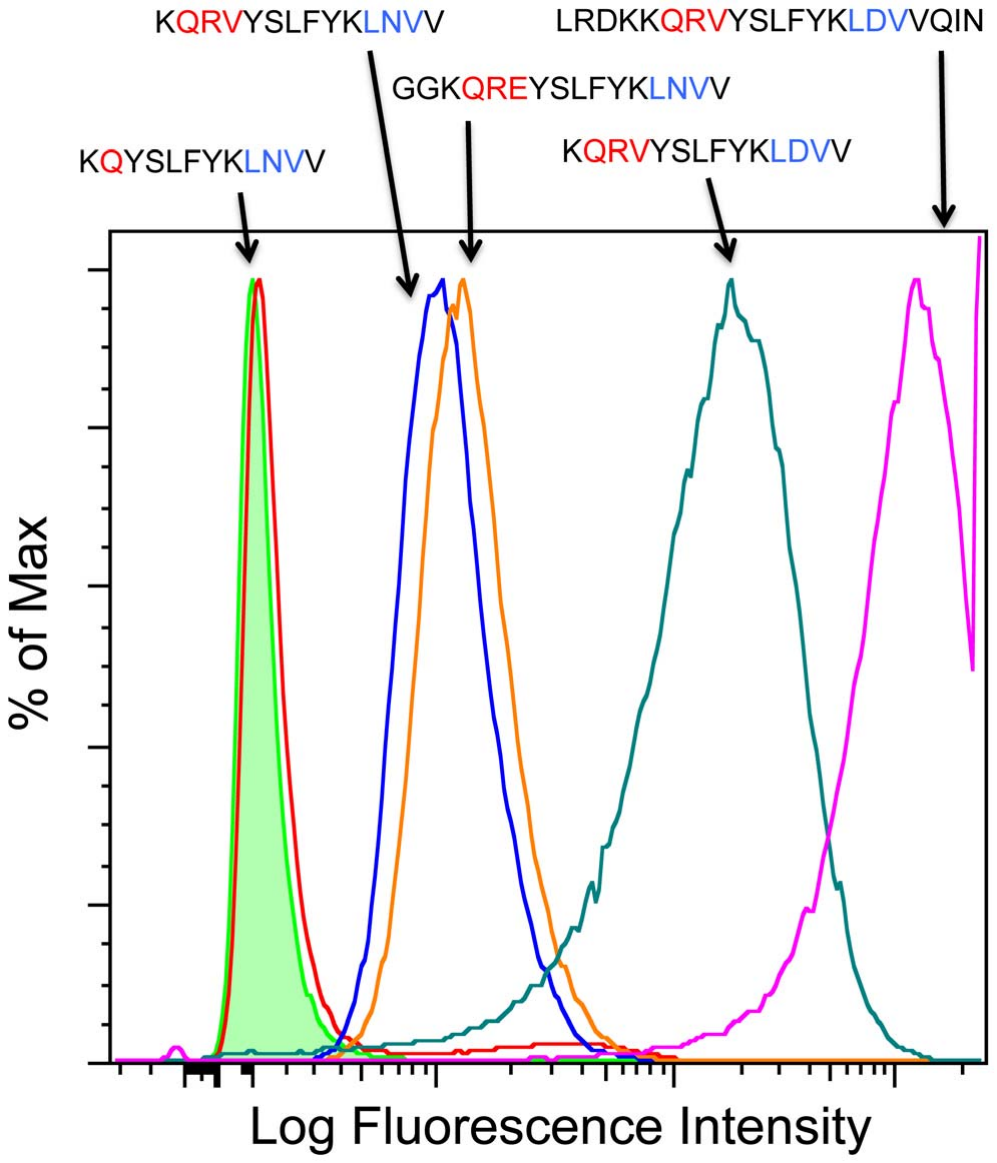
The QRV/QKE motif rescues binding of peptides with a mutated canonical α4β7 binding site. The graph shows the histogram peaks generated by binding of short biotinylated peptides to α4β7^+^ RPMI 8866 cells. The binding activity of the short peptide with a mutated α4β7 binding site (LNV instead of LDV) was restored with the addition of QRV (blue) or QKE (orange) to the N-terminus of the peptide. In the diverse set of peptides tested, maximal binding was achieved with the inclusion of both the QRV and LDV motifs (green and magenta). Green histogram is NeutrAvidin control, LFI = Log Fluorescence Intensity.

Since the engagement of the integrin binding site (LD[I/V]^179–181^) with α4β7 receptors on cells appears to depend on the QRV^170–172^ upstream sequence, we evaluated the role this upstream position plays in binding for a variety of HIV-1 viruses. We selected four different V2 peptide sequences based on chemical rather than phylogenetic diversity from the compendium of sequences of circulating HIV-1 viruses (see Methods). The sequences were chosen to include the α4β7 binding site and to begin at the loop preceding the C β-strand in the structure of the V1/V2 domain [16] (**Table 1**). Of these four sequences, only peptide 1 bound maximally to α4β7-expressing T cells (**Figure 8**). This peptide was the most polar and contained both the QRV and LDV motifs. Although peptide 2 contained one of the motifs used in the RV144 vaccine (QKE^170–172^) and peptide 3 contained one of the most common motifs in subtype AE strains (QQV^170–172^) they bound only minimally to primary T lymphocytes expressing active α4β7. Only peptide 1, containing the QRV^170–172^ motif, bound maximally to α4β7 on primary CD4^+^ T cells and CD8^+^ T cells (**Figure 8**). Although the presence of either QRV^170–172^ or LD[I/V]^179–181^ alone did not result in optimal binding, the presence of both together conferred maximal α4β7 binding. Analysis of peptides with mutated integrin α4β7 binding sites (LNV^179–181^) showed that the presence of QRV^170–172^ or QRE^170–172^ rescued integrin α4β7 binding (compare red trace with blue and orange traces in **Figure 9**). Again, this experiment showed maximal α4β7 binding for a number of diverse peptides spanning different lengths (**Figure 9**). The peptide with the strongest activity toward α4β7 (starting with sequence LRD) is also the same peptide that was previously shown to strongly react with antibodies elicited by vaccinees in the immune-correlates analysis of the RV144 trial (**Figure 9** **)** [2], [10]. In the analysis, Karasavvas et al. used ELISA to test antibody responses of RV144 participants to linear biotinylated V2 loop peptides. They found that though the peptide with sequence LRDKKQRVYSLFYKLDVVQIN (same peptide shown in **Figure 9**) binds to 84% of vaccinee plasma tested, a shorter sequence excluding the α4β7 binding domain (LRDKKQRVYSLFYK) binds to 93% of vaccinee plasma. Interestingly, when LDV^179–181^ is removed from the peptide, it still retains its reactivity with the serum of vaccinated patients [10].

## Discussion

In order to conclude that a particular HIV epitope, when mimicked in a vaccine immunogen, confers protection from infection, four criteria should be met: 1) the epitope must be immunogenic, i.e., elicit relevant antibodies upon vaccination; 2) antibodies targeted to the epitope must be correlated with reduced risk of infection, or better, they are definitively and experimentally proven to protect against virus acquisition; 3) the epitope should be reasonably conserved across different strains; and 4) the Abs targeting the epitope should have functional effects on the virus. The first two of these requirements have been established for the epitope region in the V2 loop spanning from positions 165 to 178 by prior analysis of the RV144 trial [2], [3]. Certain amino acids within this region are conserved across multiple strains and subtypes [17] and RV144 protective anti-V1V2 Abs cross-react across strains from most major subtypes [18], so the third requirement may be met by certain Abs. Since the V2^166–178^ segment technically does not include the known α4β7 integrin-binding LD[I/V]^179–181^ tripeptide, which is directly adjacent to it, the last requirement is not yet satisfied for epitopes within V2^166–178^. The α4β7 site at 179–181 is indeed a functional site, associated with gut homing and immune modulation of the host response to the virus [9]. Based on the weak neutralization activity observed with RV144 vaccinee serum, the function of V2^166–178^ directed Abs has been hypothesized to be Fc-mediated and therefore unrelated to α4β7 integrin-binding. However, we show in this report that certain amino acids within V2^166–178^ may be structurally and functionally linked to the α4β7 integrin-binding LD[I/V]^179–181^ tripeptide, thereby directly associating the Ab-targeted region of V1V2 with an HIV-host interaction function.

The challenge faced by our experimental approach was that measurements of α4β7 engagement by HIV-derived proteins/peptides/viruses are subject to many variables outside of the actual α4β7 receptor engagement site. These include post-translational modifications (PTMs), which vary widely between different HIV-1 strains, and tertiary and quaternary viral spike structural factors. For this reason, reliable classification of virions or recombinant/cell surface gp120 from different strains into α4β7 binders or non-binders is difficult. We used conformationally unconstrained short peptides with sequences identical to those found in V2^166–178^ from different HIV-1 strains in order to control for these factors. This approach was predicated on our prior observations that the conformational dynamics of the crown of the closely related V3 loop are captured by isolated short peptides, indicating that the V3 crown is apparently unrestrained by its stems connecting it to the rest of gp120 [13], [14]. The opposite study, of correlating sequence variations in the V2^166–178^ region within whole gp120 monomers or trimers with measurements of the α4β7 interaction, might not reveal the relationship we found using our short peptide approach, as PTMs, tertiary or quaternary factors may mask the dependence we detected. For example, a V2^166–178^ segment containing “QKE” but held into a strained conformation in a gp120 monomer by tertiary contacts may still bind to α4β7 *in vitro*, thus confounding the correlation we observed. Conversely, this segment may be released by trimer conformational dynamics and behave like the free peptides we have used in the true physiological scenario. If this is true, the QRV motif in the gp120 sequence from any given HIV strain might be considered a marker either of the dependence of the α4β7 interaction only on the local secondary structure of V2^166–178^ (as opposed to a yet undefined tertiary/quaternary interactions) or, *a priori*, of the presence of the α4β7 interaction itself. If the former, the suggestion would be that the strains bearing QRV are more flexible and can more easily adopt the liganded or exposed form of the trimer.

V2^166–178^ is structurally polymorphic, adopting an α–helical structure in complex with monoclonal Abs CH58 and CH59 [19] and a β-strand structure in complex with monoclonal Ab PG9 [16]. In a trimer model of V2^166–178^, this segment is highly constrained to a particular conformation by the V1V2 tertiary structure [15]. Indeed, the conformational dynamics of V2^166–178^ appear to be influenced by tripeptide QRV^170–172^, and these dynamics may determine the proper functional presentation of LD[I/V]^179–181^ for α4β7. In strain sequences that normally do not exhibit QRV^170–172^ (e.g. the A244 and MN strain sequences), the conformational dynamics are relatively narrowly constrained to a β-strand conformation. However, with the replacement of the 170–172 positions with QRV^170–172^, the peptide structure becomes more flexible/variable, forming different types of beta-like or alpha helical-like folds, some of which resemble the form recognized by CH58 and CH59 [19] (**Figure 6** **, Figures S3–S4**). Interestingly, one of the peptides tested here that strongly interacts with α4β7 (**Figure 9**) was also found to strongly interact with antibodies elicited by vaccinees in the RV144 trial immune correlates study [6], [10]. Even more telling, is that the peptide reacts with more vaccinee sera when LDV^179–181^, the α4β7 binding site is removed. Therefore, it is likely that this QRV^170–172^ tripeptide is immunogenic and elicited antibodies in the RV144 trial.

There is no reason, *a priori*, to expect that CD4/CCR5 mediated attachment and junction need to be fundamentally limited to α4β7 mediated gut homing. Indeed, there is no evidence suggesting that the α4β7 interaction overlaps with the CD4/chemokine receptor based viral attachment and entry site from a molecular point of view as the latter processes involve different locations within gp120 and different host receptors. Thus, reports that α4β7 has little effect on HIV infection *in vitro* [6] may not be relevant to the complex process of HIV acquisition and establishment *in vivo*. Alternatively, it is possible that α4β7 function ranges by subtype as the study by Parish et al. focused only on subtype C strains. Indeed, higher frequencies of α4β7^+^ T cells correlate with increased infection in a non-human primate model [20]. The most likely conclusion from the currently available evidence is thus that α4β7-mediated HIV-host interactions may be an important CCR5/CXCR-*independent* step in the establishment of HIV infection. Thus, at the present time, preventing this from occurring by eliciting antibodies to the gp120 regions associated with α4β7 integrin binding is supported as a theoretical approach to protect individuals from acquiring HIV. The RV144 immunogenic V2^166–178^ segment is directly adjacent to, but does not overlap with, the known α4β7 integrin binding LD[I/V]^179–181^ tripeptide. While steric hindrance from an Ab binding nearby could explain how V2^166–178^-directed Abs block α4β7-mediated functions, our study suggests that these Abs could directly inhibit α4β7- gp120 interactions in some viruses via targeting the functionally significant site at V2 positions 170–172.

In summary, this work supports a new protective mechanism from vaccine elicited V2 directed antibody responses and furthers our understanding of structural and functional interactions between α4β7 and gp120 in HIV infection. Future vaccine trials using envelope immunogens containing V2 and the QRV^170–172^ tripeptide can be analyzed for functional responses directed to this important region in mechanistic correlates analyses.

## Supporting Information

Figure S1 **Increased expression of activated α4β7 on purified T cells.** Flowcytometric analysis of expression of α4β7 on magnetically sorted CD4^+^ T cells before and after activation with retinoic acid for 5–7 days.
(TIF)

Figure S2 **Determining peptide polarity.** All known V2 loop sequences were obtained from the Los Alamos National Laboratories Database (30830 sequences) and filtered to select only one sequence per patient (leaving 4200 sequences). The percent polarity for each was calculated by the ratio of polar amino acids (E, D, K, R, H, S, T, N, Q) to all amino acids and plotted versus the length of each V2 sequence. The green-circled V2 loop sequence is Peptide 1 from Table 1.
(TIF)

Figure S3 ***ab initio*** **peptide folding.** Peptide 1 (the sequence used in strain A244 of the RV144 vaccine) is predicted to consistently fold into a beta hairpin. Presented are the 16 most energetically favorable conformations predicted by our software. The peptide is shown in ribbon representation with the α4β7 binding domain (LDI^179–181^) shown in ball-and stick and colored according to residue.
(TIF)

Figure S4 ***ab initio*** **peptide folding.** Peptide 1 (the sequence used in strain A244 of the RV144 vaccine) with the addition of QRV to the N-terminus of the peptide, folds into more variable conformations including beta-like and alpha helical-like folds. Presented are the 16 most energetically favorable conformations predicted by our software. The peptide is shown in ribbon representation with the α4β7 binding domain (LDI^179–181^) shown in ball-and stick and colored according to residue.
(TIF)
