# Supplementary figures and images for: Cryptic Determinant of α4β7 Binding in the V2 Loop of HIV-1 gp120
Source: PLoS One. 2014 Sep 29;9(9):e108446. doi: 10.1371/journal.pone.0108446 (PMC4180765; doi:10.1371/journal.pone.0108446)

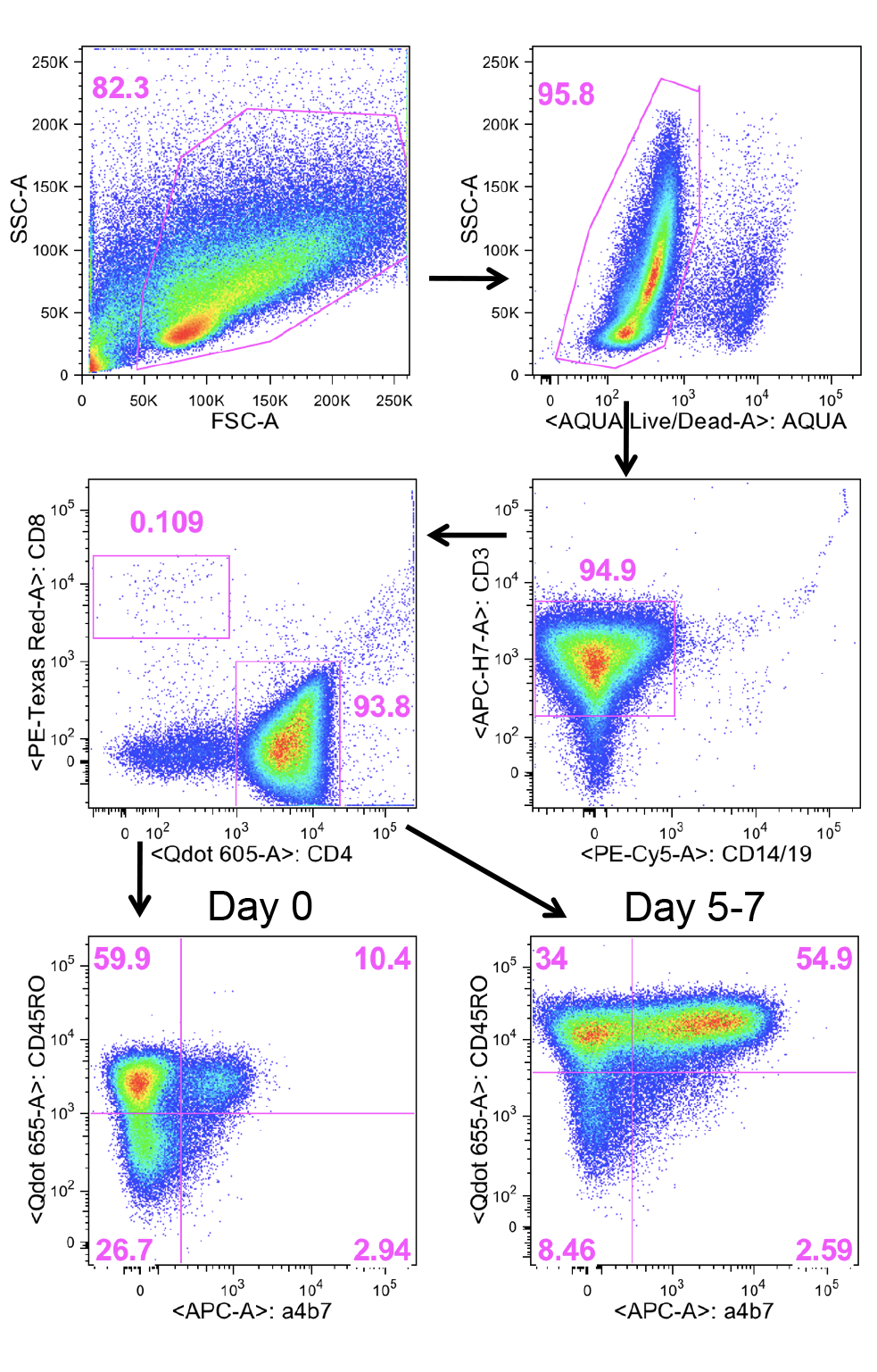

Supplement: Figure S1 — Increased expression of activated α4β7 on purified T cells. Flowcytometric analysis of expression of α4β7 on magnetically sorted CD4+ T cells before and after activation with retinoic acid for 5–7 days. (TIF) [file pone.0108446.s001.tif]

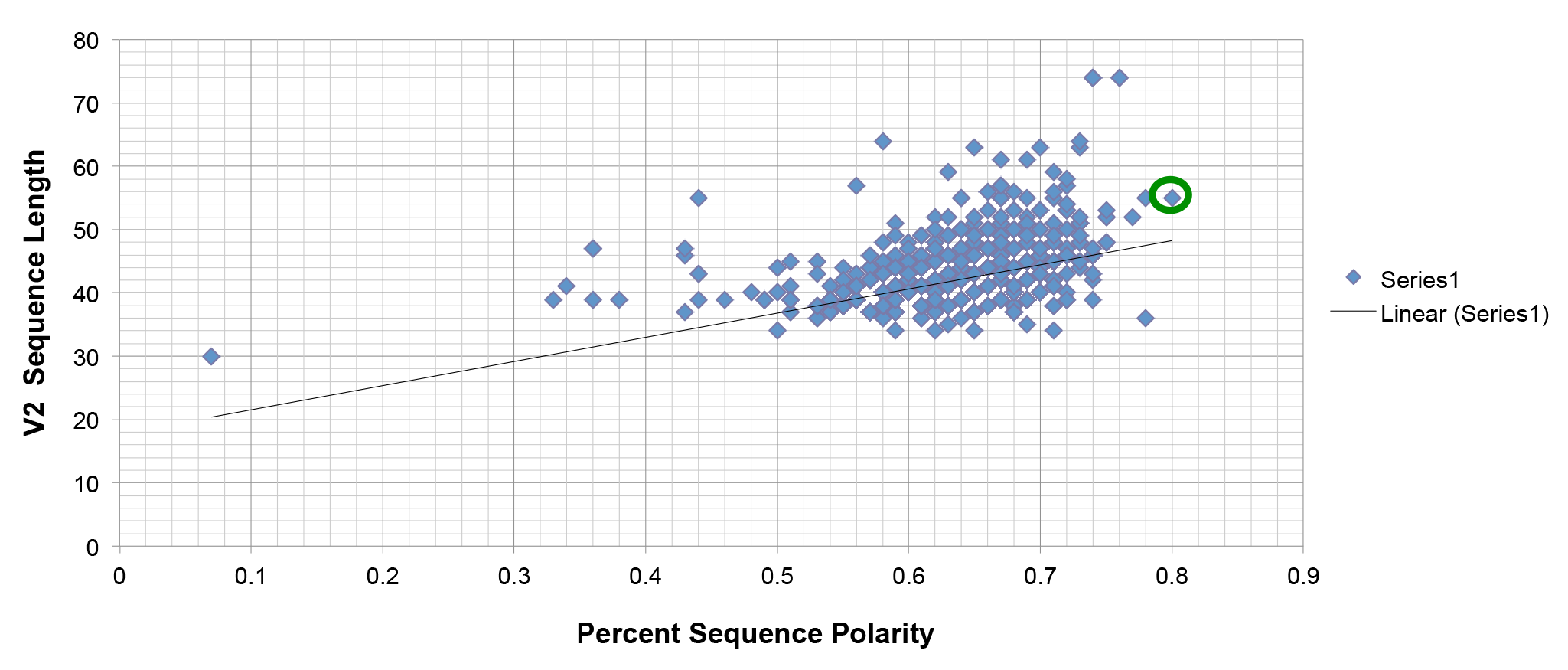

Supplement: Figure S2 — Determining peptide polarity. All known V2 loop sequences were obtained from the Los Alamos National Laboratories Database (30830 sequences) and filtered to select only one sequence per patient (leaving 4200 sequences). The percent polarity for each was calculated by the ratio of polar amino acids (E, D, K, R, H, S, T, N, Q) to all amino acids and plotted versus the length of each V2 sequence. The green-circled V2 loop sequence is Peptide 1 from Table 1. (TIF) [file pone.0108446.s002.tif]

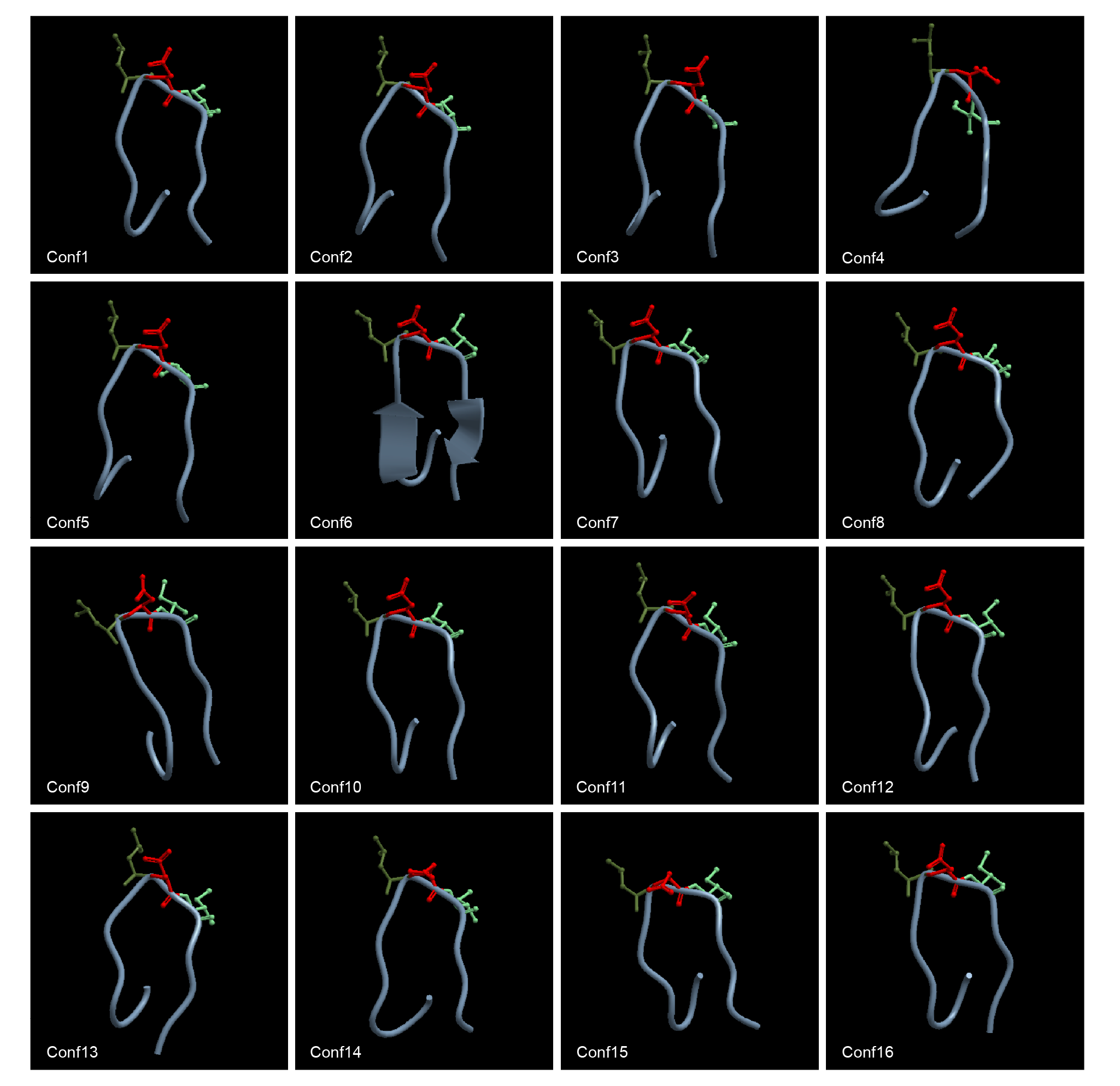

Supplement: Figure S3 — ab initio peptide folding. Peptide 1 (the sequence used in strain A244 of the RV144 vaccine) is predicted to consistently fold into a beta hairpin. Presented are the 16 most energetically favorable conformations predicted by our software. The peptide is shown in ribbon representation with the α4β7 binding domain (LDI179–181) shown in ball-and stick and colored according to residue. (TIF) [file pone.0108446.s003.tif]

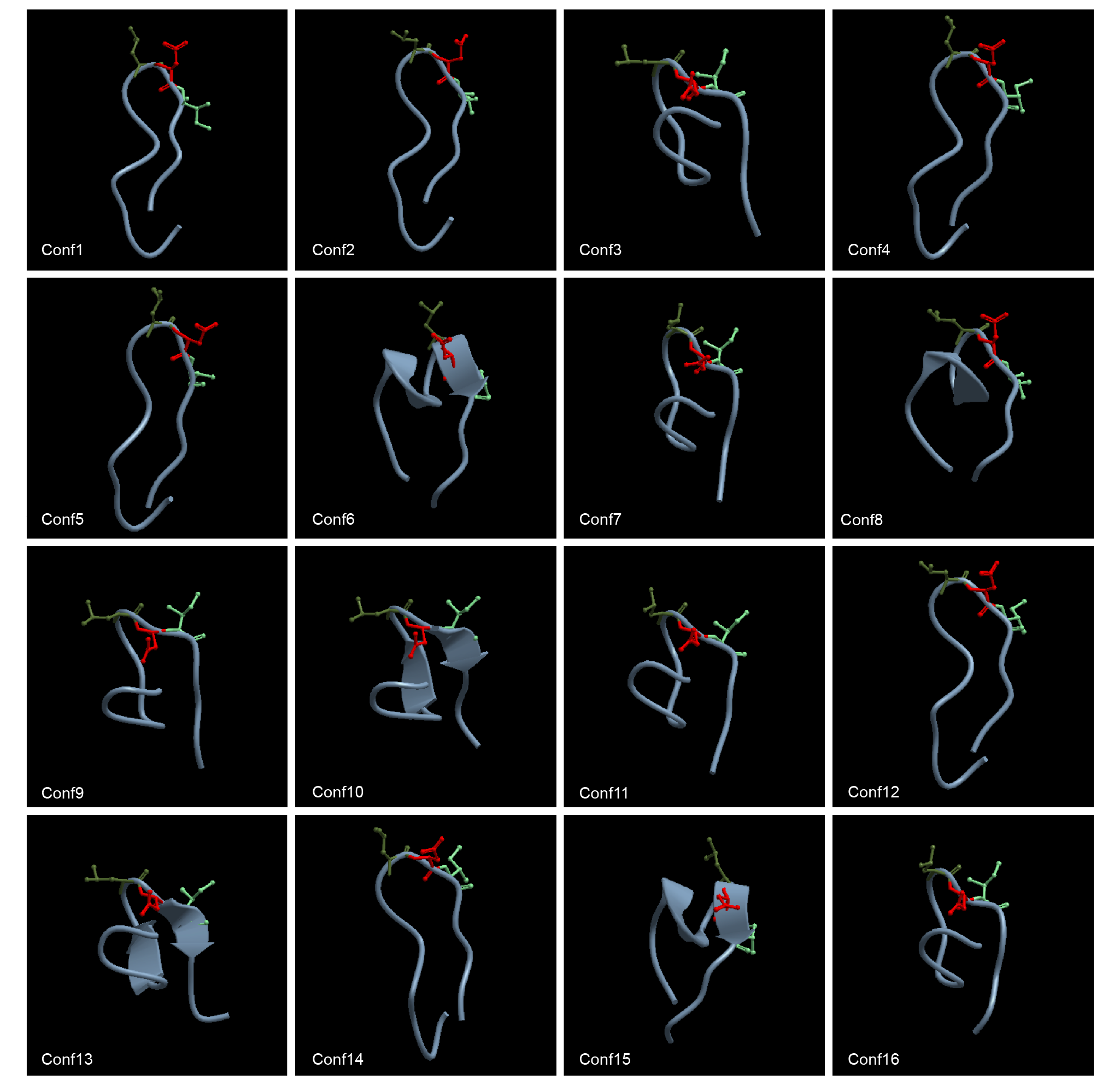

Supplement: Figure S4 — ab initio peptide folding. Peptide 1 (the sequence used in strain A244 of the RV144 vaccine) with the addition of QRV to the N-terminus of the peptide, folds into more variable conformations including beta-like and alpha helical-like folds. Presented are the 16 most energetically favorable conformations predicted by our software. The peptide is shown in ribbon representation with the α4β7 binding domain (LDI179–181) shown in ball-and stick and colored according to residue. (TIF) [file pone.0108446.s004.tif]
